# Effect of freely chosen vs. a scientifically based nutritional strategy on marathon race performance

**DOI:** 10.1186/1550-2783-10-S1-P8

**Published:** 2013-12-06

**Authors:** Ernst A Hansen, Anders Emanuelsen, Robert M Gertsen, Simon SR Sørensen

**Affiliations:** 1 Department of Health Science and Technology, Aalborg University, DK-9220 Aalborg, Denmark; 2 Center for Sensory-Motor Interaction (SMI), Aalborg University, DK-9220 Aalborg, Denmark

## Background

Based on laboratory studies performed through decades, it is suggested that carbohydrate intake during prolonged exercise improves performance. However, we do not know much about whether marathon race performance in practice can be improved by intervening with a scientifically based nutritional strategy. The aim of the study was to test the hypothesis that a marathon race can be completed faster by applying a scientifically based nutritional strategy than by applying a freely chosen nutritional strategy.

## Methods

Twenty-eight non-elite marathon runners (age: 37.7 ± 9.6 years, height: 180.8 ± 10.6 cm, body mass: 77.0 ± 13.1 kg) performed a 10 km running time trial seven weeks before Copenhagen Marathon 2013, and in addition stated their self-expected finishing time in the upcoming race. Based on the first of these two variables of pre-race estimated marathon running ability, runners were divided into two groups that subsequently performed the marathon race on, respectively, a freely chosen (FREE) and a scientifically based (SCI) nutritional strategy. A matched pairs design was applied. Thus, before the race, the runners in the two groups were paired based on their pre-race 10 km running time trial time. SCI consisted of a combined intake of energy gels and water. One energy gel contained 20 g glucose, 0.02 g sodium, and 0.03 g caffeine. Two gels should be consumed with 200 ml water, 10-15 min before the start of the race. The next gel should be consumed 40 min after the start of the race. Subsequently, one gel should be consumed every 20th min throughout the remainder of the race. In addition to the energy gels, a water intake of 750 ml per hour was recommended. In total, the target intake in SCI amounted to approximately 750 ml water, 60 g glucose, 0.06 g sodium, and 0.09 g caffeine pr. hour.

## Results

The pre-race estimation of running ability revealed similar 10 km running time trial times for runners applying FREE and SCI [2740 ± 272 (min-max: 2295 - 3301) s and 2744 ± 277 (min-max: 2272 - 3301) s, respectively, p=.25]. Self-expected finishing times were also similar for runner applying FREE and SCI [224.6 ± 24.7 (min-max: 175 - 285) min and 219.9 ± 25.3 (min-max: 172 - 250) min, respectively, p=.32]. Measured finishing time in Copenhagen Marathon 2013 amounted to 229.4 ± 25.1 (min-max: 183.3 - 289.0) min for runners applying FREE and 218.5 ± 24.9 (min-max: 168.4 - 273.5) min for runners applying SCI (p=.01).

## Conclusion

It was concluded that non-elite marathon runners on average completed a marathon race approximately 5% faster by applying a scientifically based nutritional strategy than by applying a freely chosen nutritional strategy.

